# Overdominant expression of genes plays a key role in root growth of tobacco hybrids

**DOI:** 10.3389/fpls.2023.1107550

**Published:** 2023-01-31

**Authors:** Kai Pi, Ying Huang, Wen Luo, Shuaibo Zeng, Zejun Mo, Lili Duan, Renxiang Liu

**Affiliations:** ^1^ College of Tobacco, Guizhou University, Guiyang, China; ^2^ Key Laboratory of Tobacco Quality in Guizhou Province, Guiyang, China; ^3^ College of Agriculture, Guizhou University, Guiyang, China

**Keywords:** heterosis, overdominant expression, root system, transcriptomics, WGCNA

## Abstract

Heterosis has greatly improved the yield and quality of crops. However, previous studies often focused on improving the yield and quality of the shoot system, while research on the root system was neglected. We determined the root numbers of 12 F_1_ hybrids, all of which showed strong heterosis, indicating that tobacco F_1_ hybrids have general heterosis. To understand its molecular mechanism, we selected two hybrids with strong heterosis, GJ (G70 × Jiucaiping No.2) and KJ (K326 × Jiucaiping No.2), and their parents for transcriptome analysis. There were 84.22% and 90.25% of the differentially expressed genes were overdominantly expressed. The enrichment analysis of these overdominantly expressed genes showed that “Plant hormone signal transduction”, “Phenylpropanoid biosynthesis”, “MAPK signaling pathway - plant”, and “Starch and sucrose metabolism” pathways were associated with root development. We focused on the analysis of the biosynthetic pathways of auxin(AUX), cytokinins(CTK), abscisic acid(ABA), ethylene(ET), and salicylic acid(SA), suggesting that overdominant expression of these hormone signaling pathway genes may enhance root development in hybrids. In addition, *Nitab4.5_0011528g0020、Nitab4.5_0003282g0020、Nitab4.5_0004384g0070* may be the genes involved in root growth. Genome-wide comparative transcriptome analysis enhanced our understanding of the regulatory network of tobacco root development and provided new ideas for studying the molecular mechanisms of tobacco root development.

## Introduction

The growth period of tobacco is about 120 to 130 days. The plant is tall and needs a lot of water and nutrients for growth and development, and these substances are transported to the shoot *via* the root system (Zhaojun, 2021). The root system is an indispensable organ for tobacco growth and consists of three parts: main root, lateral root, and adventitious root. Many lateral roots can produce secondary lateral roots and tertiary lateral roots, which become the main part of the root system (Yang et al., 2002). Tobacco roots, like most plant roots, have the functions of absorbing and transporting soil moisture and inorganic salts, fixing and supporting plants, and synthesizing amino acids and proteins (Meister et al., 2014), which have a great impact on the growth and development of tobacco plants and the yield and quality of tobacco leaves. In addition, the iconic tobacco product, nicotine, is synthesized in the root system and then transported to the leaves (Solt, 1959) and is extremely important for the smoking quality of tobacco (Tong et al., 2020). Heterosis is a common natural phenomenon, which refers to when heterozygotes generated by hybridization are superior to the two parents in terms of growth potential, biomass, stress resistance, and adaptability (Shull, 1908). In most previous studies, heterosis is usually demonstrated in terms of biomass, yield, and stress resistance (Fujimoto et al., 2018; Li et al., 2021). In recent years, however, some studies have shown that the level of heterosis in root traits may be higher than that in the overground part (Ju et al., 2018; Dafna et al., 2021), and some efforts have been made to reveal the molecular mechanism of heterosis in root development (Paschold et al., 2010; Shalby et al., 2021), which means that the root system may be the perfect organ to study the genetic basis of tobacco heterosis. Although there are several genetic models used to describe heterosis, including dominant, overdominant, and epistasis, but its molecular basis remains poorly understood (Chen, 2013). To better understand heterosis, Thiemann et al. (2014) proposed two models of gene expression correlation: additive and non-additive expression patterns. In different crops, gene expression shows different additive and non-additive expression patterns (Tian et al., 2018; Shahzad et al., 2020; Shalby et al., 2021). In recent years, a large number of transcriptome research advances have provided new insights into the molecular basis of heterosis in species such as *Arabidopsis* (Fujimoto et al., 2012; Wang et al., 2019; Liu et al., 2020), rice (Shao et al., 2019; Ren et al., 2020), maize (Ko et al., 2016), and rape (Xiong et al., 2022).

Root development is the result of the synergistic action of various plant hormones. Hormones play an important role in root development through regulation of gene expression (Jia et al., 2022). Auxin (AUX), one of the most important plant hormones, regulates the growth of primary and lateral roots by promoting cell division and elongation (Rivas et al., 2022). Furthermore, cytokinins (CTKs) have been reported to have important control roles in the regulation of root structure and nutrients (Muraro et al., 2011). Abscisic acid (ABA) has an important control role in regulating root adaptive responses (Belda-Palazon et al., 2018; Ma et al., 2018). Ethylene (ETH) plays an important control role in root cell elongation (Song et al., 2019). Salicylic acid (SA) controls root growth by regulating the activity of root apical meristem (Bagautdinova et al., 2022), and studies have shown that *Arabidopsis* hybrids with superior SA content exhibit root growth heterosis (Zhang et al., 2016). The research shows that the number of root cells, cell differentiation and other processes will affect the root development (Cuadrado et al., 1987). Hormone content can also affect root development by affecting the number of root cells, cell differentiation and other processes (McCarthy-Suárez, 2021).

In tobacco, heterosis can be used to obtain tobacco hybrids with higher nicotine and potassium contents than the parents, and our previous studies have shown that nicotine heterosis is due to the higher efficiency of nicotine synthesis in hybrids (Tian et al., 2018; Mo et al., 2021). Since nicotine is synthesized in the root system (Solt, 1959), tobacco roots may be the basis for the heterosis of nicotine and potassium contents, which directly affects the development and quality of tobacco leaves. Therefore, this study preliminarily revealed the expression of early root heterosis in tobacco and performed transcriptome sequencing of two hybrids with strong heterosis to analyze the differentially expressed genes (DEGs), gene expression patterns, and biological processes that mediate heterosis in root growth. These new findings may help to reveal the biological mechanism of tobacco root heterosis and discover related candidate genes. These findings may also be significant in guiding the selection of new tobacco germplasm with high yield and high quality.

## Materials and methods

### Plant materials, growth conditions, and sample preparation

Based on our previous research (Tian et al., 2018; Pi et al., 2022), we selected the hybrids and their parents with different nicotine and potassium contents, and 12 hybrids were made from 7 parents according to the incomplete diallel crossing (NCII) method(**Supplementary File 1, Table 1**). All materials were provided by Guizhou Key Laboratory of Tobacco Quality Research. From 2021-2022, in the tobacco research base of Guizhou University, Yangwu Township, Anshun City, Guizhou Province, China, Seeds were sown in greenhouses using the floating seedling method, and the seedlings with six real leaves were sampled. The methods were as follows: Firstly, 3 seedlings with similar growth vigor were selected for root phenotype determination. After that, fresh biological samples were frozen with liquid nitrogen and stored in an ultra-low temperature refrigerator at - 80 ℃. These fresh samples were used for transcriptome sequencing analysis and RT-qPCR.

### Determination of root phenotype and calculation of heterosis

The cleaned complete root system was scanned with the root scanner LA2400 to obtain a clear image of the root structure. Use WinRhizo Pro 2021a software (Regent, Canada) to count tobacco root tips.

### RNA isolation and sequencing

Total RNA was extracted from the root tissue using TRIzol^®^ Reagent (Plant RNA Purification Reagent for plant tissue) according to the manufacturer’s instructions (Invitrogen), and genomic DNA was removed using DNase I (TaKara). Then RNA quality was determined by 2100 Bioanalyser (Agilent) and quantified using the ND-2000 (NanoDrop Technologies). Only high-quality RNA sample (OD260/280 = 1.8~2.2, OD260/230≥2.0, RIN≥6.5, 28S:18S≥1.0, >1μg) was used to construct sequencing library. Paired-end RNA-seq sequencing library was sequenced with the NovaSeq 6000 sequencer (2 × 150bp read length).

### Transcriptomics data processing and analysis

By preprocessing the raw reads, short sequences with a length <25 nt and low quality sequences were removed. After preprocessing, the obtained reads were mapped to the *Nicotiana tabacum* sequenced cultivar K326 genome (Edwards et al., 2017) using the splice-aware mapping tool, Tophat2 (Kim et al., 2019). RSEM was used to quantify gene abundances (Li and Dewey, 2011). Essentially, differential expression analysis was performed using the DESeq2 (Love et al., 2014), DEGs with |log2FC| ≥ 1 and p ≤ 0.05 were considered to be significantly different expressed genes. Gene ontology (GO) functional-enrichment analysis were carried out by Goatools (Klopfenstein et al., 2018). In addition, we use PlantTFDB (http://planttfdb.gao-lab.org/) Transcription factor analysis of genes.

### Classification of gene expression patterns

In order to divide the differentially expressed genes into 12 expression modes, according to the definition of Rapp et al. (2009). we used STEM software (Ernst and Bar-Joseph, 2006) to perform additional analysis on the differentially expressed genes. To allow clustering on a reasonable number of possible model profiles, the parameter for “STEM clustering method,” model profiles “was set to 50 and 2 was selected as the” maximum unit change between time points “, and” Minimum Absolute Expression Change “was set to 0 to classify the expression patterns of all differentially expressed genes.

### Identification of core genes in tobacco root development

The typical genes of Arabidopsis root development were used to identify core genes in tobacco. BLASTP identified 6 core genes with E-values truncated at 1E-10. To further classify root development genes in tobacco, phylogenetic analyses were performed using homologous protein sequences from *Arabidopsis thaliana* and *Triticum aestivum* L. The phylogenetic tree was constructed by MEGA 11.0(https://www.megasoftware.net/), The ultrafast bootstrap with 1000 replicates was conducted to obtain the supporting values for each bootstrap of the tree. Finally, gene trees were visualized and colored using iTOL(https://itol.embl.de/).

### RT-qPCR validation

For the validation of the transcriptome data, we randomly selected 6 DEGs for RT-qPCR (PCR quantitative real-time) analysis. **Supplementary File 1: Table 16** lists the genes and corresponding primers used in **RT-**qPCR. The total RNA used in the sequencing was reversely transcribed to obtain cDNA, which was used as a template to amplify the target genes, and the RT-qPCR experiment was conducted. The RNA was reversely transcribed into cDNA using the FastKing reverse transcription kit (Tiangen, China) as per the manual’s instructions. qPCR was performed using the BIO-RAD CFX96 Real-Time qPCR system. The relative expression of each gene was calculated using 2^−△△Ct^ (Livak and Schmittgen, 2001).

### Statistical analysis

SPSS 25.0 software was used for statistical analyses. The variance analysis of root phenotype was carried out using the Duncan’s new multiple range method (P < 0.05). Based on the root phenotype, the values of mid-parent heterosis (MPH) were calculated according to the following method, $\text{MPH }\left(\%\right)=\left(\frac{{\text{F}}_{1}-\text{MP}}{\text{MP}}\right)\times 100$ , where F_1_represents the value of the first generation of hybrid and MP represents the average value of parents $\left(\frac{{\text{P}}_{1}+{\text{P}}_{2}}{2}\right)$ .

## Results

### Heterosis in the root number of F1 hybrids

**Figure 1:**
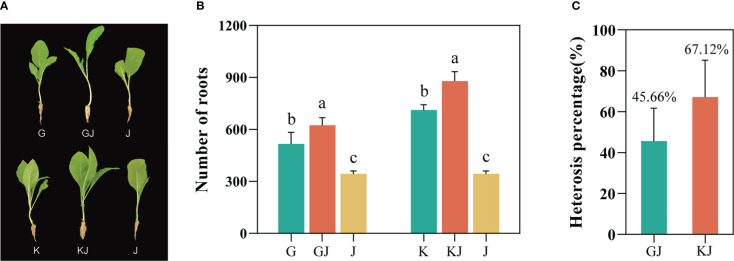
Phenotypic parameters and Heterosis of two tobacco F1 hybrids and their parents. **(A)** Photographs of the two hybrids and their parents used in this study. **(B)** Root number of F1 hybrid and its parents(G = G70, J = Jiucaiping No.2, GJ = G70 × Jiucaiping No.2, K = K326, KJ =K326 × Jiucaiping No.2). **(C)** Heterosis of two tobacco F1 hybrids (GJ and KJ). Error bars indicate standard error. Different small letters showed significant differences (P<0.05).

Based on the screening results of tobacco root number heterosis (**Supplementary File 1, Table 1**), the root numbers of the 12 F_1_ hybrids all showed strong heterosis values, indicating that tobacco F_1_ hybrids have general heterosis. Two highly dominant F_1_ hybrids, GJ (G70 × Jiucaiping No.2) and KJ (K326 × Jiucaiping No.2), were selected from these F_1_ hybrids for further analysis (their median parent values were not significantly different). At the tobacco seedling stage, we observed that the root development of the two hybrids were significantly better than that in their parents (**Figure 1A**). The number of root systems in GJ was 623, whereas G and J had 515 and 343, respectively; the number of roots in KJ was 878, whereas K and J had 711 and 343, respectively. The results showed that the average number of roots of both hybrids were significantly higher than that of their parents (**Figure 1B**), and the heterosis was 45.66% (GJ) and 67.12% (KJ) (**Figure 1C**).

### Global transcriptome analysis of heterosis in root number

**Figure 2:**
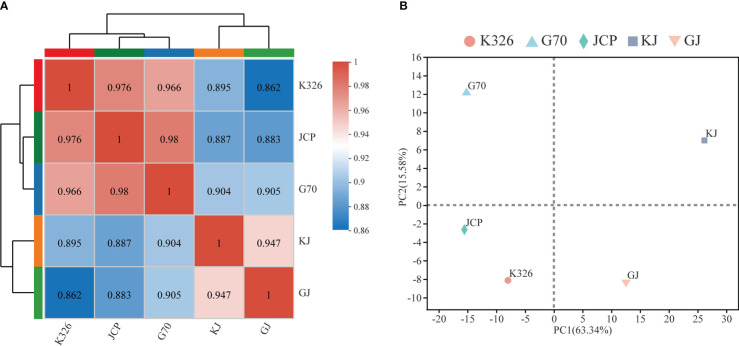
Global gene expression profiling during root development. **(A)** Pearson correlation analysis of the two hybrids and their parents, and the color code on the right represents Pearson correlation coefficient. **(B)** Principal component analysis (PCA) of RNA-Seq data. Each point shows the average of three repeats.

Using two hybrids, GJ and KJ, with strong heterosis and their three parents as sequencing materials, 15 cDNA libraries were constructed and sequenced on the Illumina Novaseq 6000 platform. Each sample generated 76-98 million clean reads in RNA-Seq, which were then used for further analysis. Clean reads were mapped to the K326 genome, and about 95% of clean reads could be mapped to the genome (**Supplementary File 1, Table 2**). A total of 39,345 genes were expressed in 15 analyzed samples (**Supplementary File 1, Table 3**). Among them, 8.30% of the genes were very highly expressed (FPKM ≥ 50), 64.19% and 27.51% of the genes were moderately (1 ≤ FPKM ≤ 10) and highly expressed (10 ≤ FPKM ≤ 50), respectively. These results reflect that there is no significant difference in expression coverage between hybrids and parents during development. However, according to hierarchical clustering analysis(**Figure 2A**) and principal component analysis of parents and hybrids (**Figure 2B**), both parents are closely clustered, and hybrids can be clearly divided into two different expression situations. These results show that there are great differences in gene expression between parents and hybrids during root development.

### Transcriptome differences between roots of hybrids and their parents

**Figure 3:**
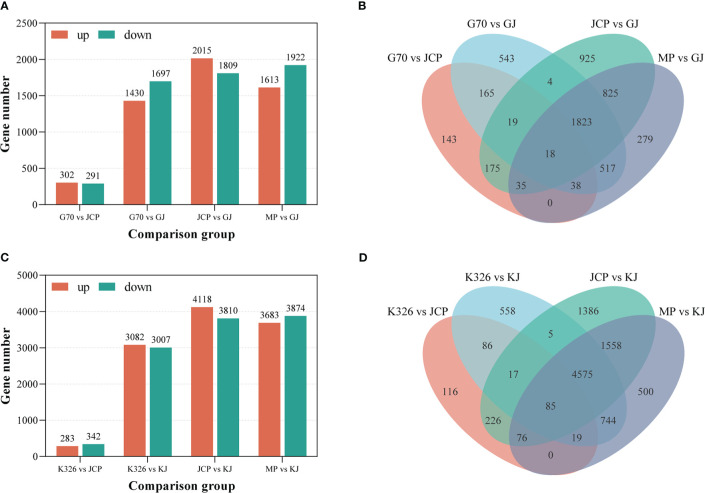
Distribution of differentially expressed genes (DEGs) between hybrids and parents. MP represents the average value of parents. **(A)** The number of different expressed genes between GJ and its parents during root development. **(B)** Venn shows the distribution of GJ and parental DEGs. **(C)** The number of different expressed genes between KJ and its parents during root development. **(D)** Venn shows the distribution of KJ and parental DEGs.

In the case of significance level P ≤ 0.05 and | log_2_ fold change | ≥ 1, we identified 1430 genes up-regulated and 1697 genes down-regulated between G (female parent) and GJ (hybrid), 2015 genes up-regulated and 1809 genes down-regulated between J (male parent) and GJ (hybrid) (**Figures 3A, B**; **Supplementary File 1, Table 4**), 1613 genes up-regulated and 1922 genes down-regulated between the middle parent and GJ hybrid, and 302 genes were up-regulated and 291 genes were down-regulated between G and J (**Supplementary File 1, Table 5**). We identified 3082 genes up-regulated and 3007 genes down-regulated between K (female parent) and KJ (hybrid), 4118 genes up-regulated and 3810 genes down-regulated between J (male parent) and KJ (hybrid) (**Figures 3C, D**), 3683 up-regulated genes and 3874 down-regulated genes between middle parent and KJ hybrid, and 283 genes up-regulated and 342 genes down-regulated between K and J **(** **Supplementary File 1, Table 6**). This demonstrates that there are relatively few differential genes between the parents of the two strong heterosis hybrids, but there are a large number of differential genes between the parents and the hybrids, which may be the reason for root number heterosis.

### F_1_ hybrid showed a overdominant gene expression pattern

**Figure 4:**
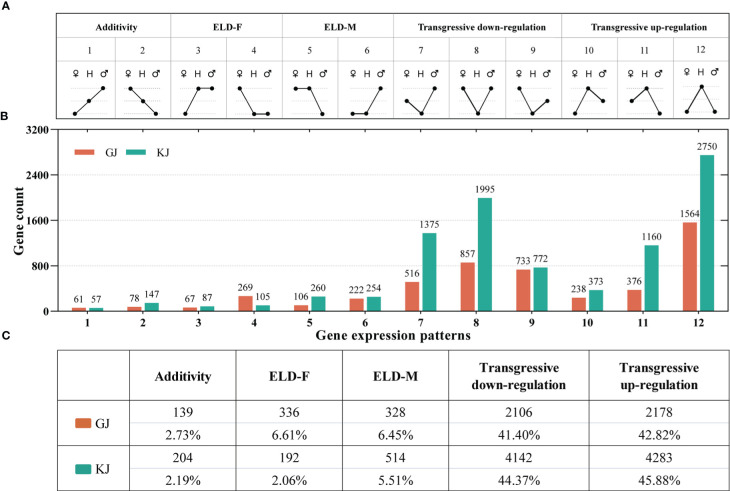
12 gene expression patterns of DEGs between hybrids and parents. **(A)** Classification of 12 gene expression patterns (♂: paternal; H: hybrid; ♀: maternal). **(B)** The number of genes of GJ and KJ hybrids in each model. **(C)** The number and proportion of the five total expression patterns after classification in DEGs.

To further analyze the DEGs of hybrids and parents, the genes were divided into 12 expression patterns (P1-P12, **Figure 4A**) according to the definition of Rapp et al. (2009). The genes in the P1 and P2 modes are additive, P3-P6 modes are dominant, and P7-P12 modes show overdominant expression, in which the genes in P7-P9 are down-regulated overdominant and P10-P12 are up-regulated overdominant. Among the overdominant genes, 238, 376, and 1564 genes of GJ hybrid showed up-regulated overdominant expression patterns(**Supplementary File 1, Table 7**), and 516, 857, and 733 genes showed down-regulated overdominant expression patterns; there were 373, 1160, and 2750 genes in KJ hybrid that showed up-regulated overdominant expression patterns(**Supplementary File 1, Table 8**), and 1375, 1995, and 772 genes that showed down-regulated overdominant expression patterns (**Figure 4B**). Among these non-additively expressed genes (P3-P12), GJ hybrid had the highest proportion (84.22%) in the expression pattern of overdominant (P7-P12), and KJ hybrid had the highest proportion (90.25%) in the expression pattern of overdominant (P7-P12) (**Figure 4C**). Therefore, our results show that the overdominant expression advantage is the main reason for the formation of heterosis of tobacco roots.

### Enrichment analysis of overdominant genes

Gene Ontology (GO) enrichment analysis was performed on the overdominant expression gene sets in GJ and KJ hybrids. We found that most overdominant genes were involved in “biological process” (55.3 – 56.1%). In GJ hybrids, most of them were significantly enriched in the jasmonic acid biosynthetic process, riboflavin metabolic process, riboflavin biosynthetic process, aminoglycan metabolic process, and jasmonic acid metabolic process (**Supplementary File 1, Table 9**; *P* < 0.05). In KJ hybrids, the overdominant genes were significantly enriched in the glucosamine containing compound catabolic process, chitin metallic process, amino sugar catabolic process, chitin catabolic process, and aminoglycan catabolic process (**Supplementary File 1, Table 10**; *P* < 0.05). According to the GO enrichment analysis of GJ and KJ co overdominant expression genes, most of the genes involved in cell differentiation, proliferation, cell growth and other processes were significantly enriched. It indicated that the overdominant expression of genes in the process of cell differentiation and proliferation was related to the formation of root heterosis.

**Figure 5:**
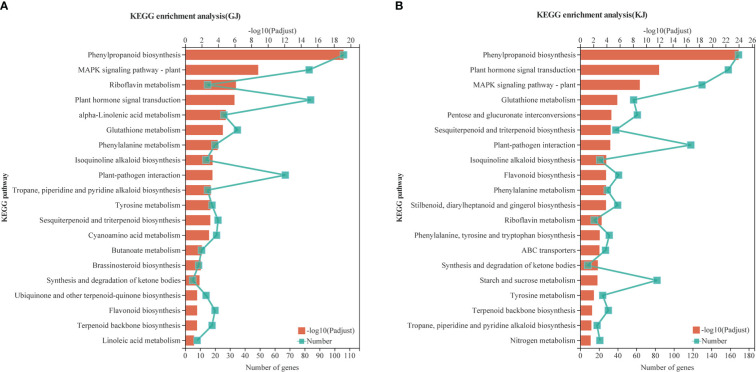
Enrichment analysis of KEGG pathway of GJ and KJ overdominant expression genes. **(A)** KEGG analysis of overdominant up-regulated and down-regulated genes in GJ hybrids. **(B)** KEGG analysis of overdominant up-regulated and down-regulated genes in KJ hybrids.

According to the analysis of Kyoto Encyclopedia of Genes and Genomes (KEGG) metabolic pathways, 4284 overdominant genes in GJ were annotated to 117 pathways (**Supplementary File 1, Table 11**; *P* < 0.05), whereas 8425 dominant genes in KJ were annotated to 123 pathways (**Supplementary File 12, Table 12**; *P* < 0.05). As shown in **Figures 5A, B**, the overdominant gene enrichment pathways of the two hybrids are mainly “phylpropanoid biosynthesis” (9.72 and 5.27%, respectively), “plant hormone signal transduction” (4.97 and 4.93%, respectively), “MAPK signaling pathway - plant” (4.91 and 4.06%, respectively), and “start and cross metadata” (2.48 and 2.56%, respectively). In addition, 84 and 158 overdominant genes of the two hybrids were related to “plant hormone signal transduction”, and 42 and 82 genes were related to “starch and sucrose metabolism”. Based on KEGG analysis, we further studied specific functional genes related to root development.

### Overdominant expression of genes related to phytohormone signaling pathway during root development

**Figure 6:**
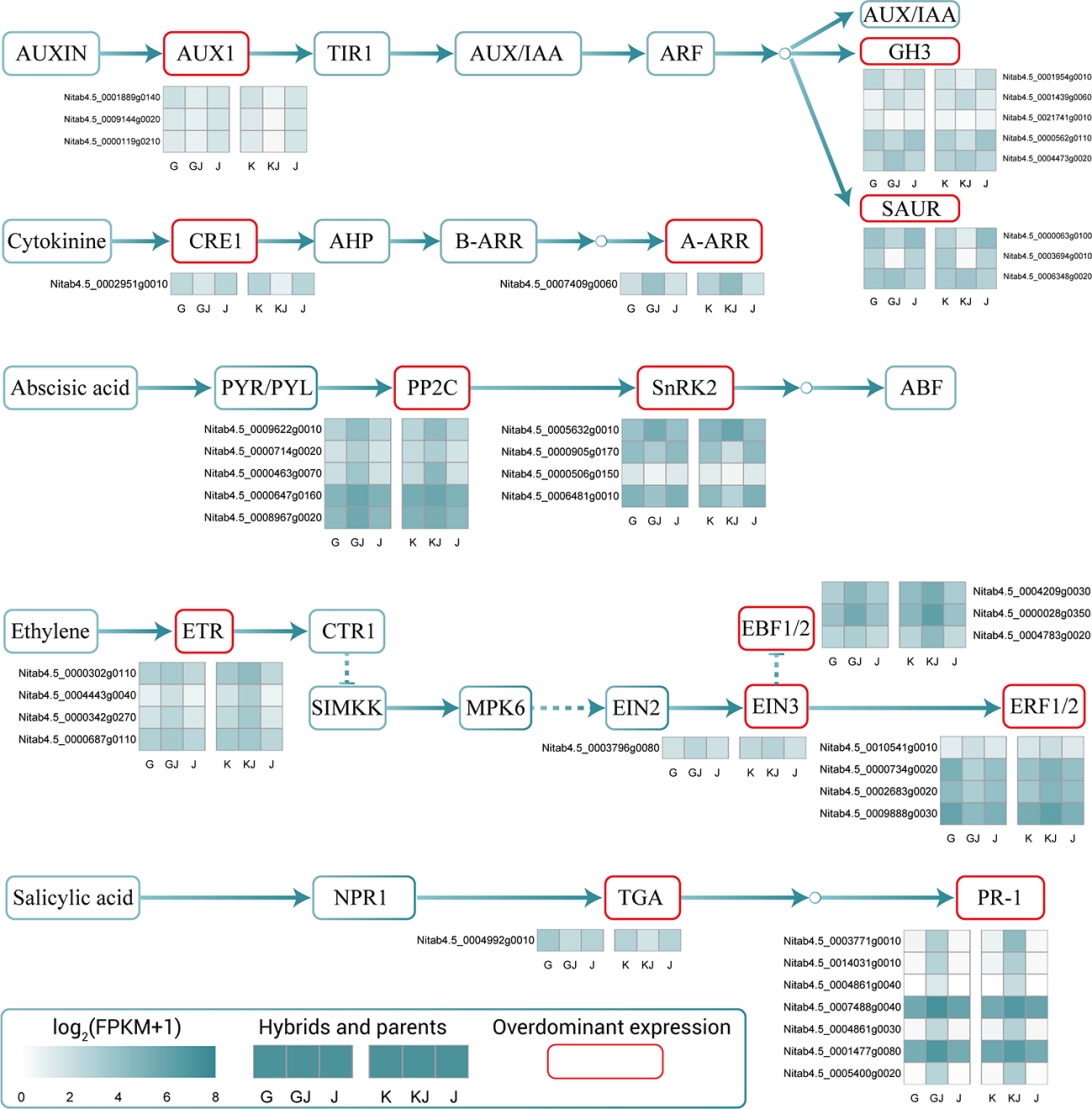
Overdominant expression genes of hormone signal transduction related to AUX, CTK, ABA, ET and SA biosynthesis. These genes showed overdominant expression in both hybrids.

Combined with functional analysis, in order to strengthen our understanding of hormone signaling during heterosis development in tobacco root growth, we focused on the overdominant genes of hormone signal transduction related to the biosynthesis of AUX, CTK, ABA, ET, and SA (**Figure 6**; **Supplementary File 1, Table 13**). In the AUX signal transduction pathway, a total of 11 genes were overexpressed in F_1_ hybrids (GJ and KJ), including 3 that were annotated to the *AUX1* gene, 5 that were annotated to the *GH3* gene, and 3 that were annotated to the AUX response gene *SAUR.* In the CTK signal transduction pathway, one receptor histidine kinase (*CRE1*) gene was negatively overexpressed in F_1_ hybrids (GJ and KJ). One gene was annotated to A-type response regulator (*A-ARR*), which showed up-regulated overdominant expression in hybrids. In the ABA signal transduction pathway, 9 genes were found to be overexpressed in F_1_ hybrids, including 5 that were annotated to protein phosphatase (*PP2C*) and 4 encoding Subcrosse non-fermenting 1-related protein kinase 2 (*SnRK2*). Interestingly, the genes related to *PP2C* showed up-regulated overdominant expression in both hybrids, of which 2 genes were highly expressed (10 ≤ FPKM ≤ 50). In the ET signal transduction pathway, 4 genes were annotated to ET receptor sensor (*ETR2*), one was annotated to *EIN3*, three were annotated to bind F-box protein 1/2 (*EBF1/2*), and four were annotated to ET responsive transcription factor 1/2 (*ERF1/2*). These ET signal transduction pathway results show that the genes related to *ETR2*, *EIN3* and *EBF1/2* are up-regulated and overdominantly expressed in both hybrids. For the SA signal transduction pathway, a total of 11 genes were overdominantly expressed in F_1_ hybrids (GJ and KJ). Among them, 1 gene was annotated to TGA transcriptional regulator, which showed down-regulated overdominant expression in the roots of hybrids, and 7 genes were annotated to protein *PR-1*. It is worth noting that these 7 *PR-1* related genes not only showed an overdominant expression pattern in the hybrid, but also had high differential expression multiples and highly expressed (FPKM ≥ 50).

### Overdominant expression of genes related to cell development during root development

**Figure 7:**
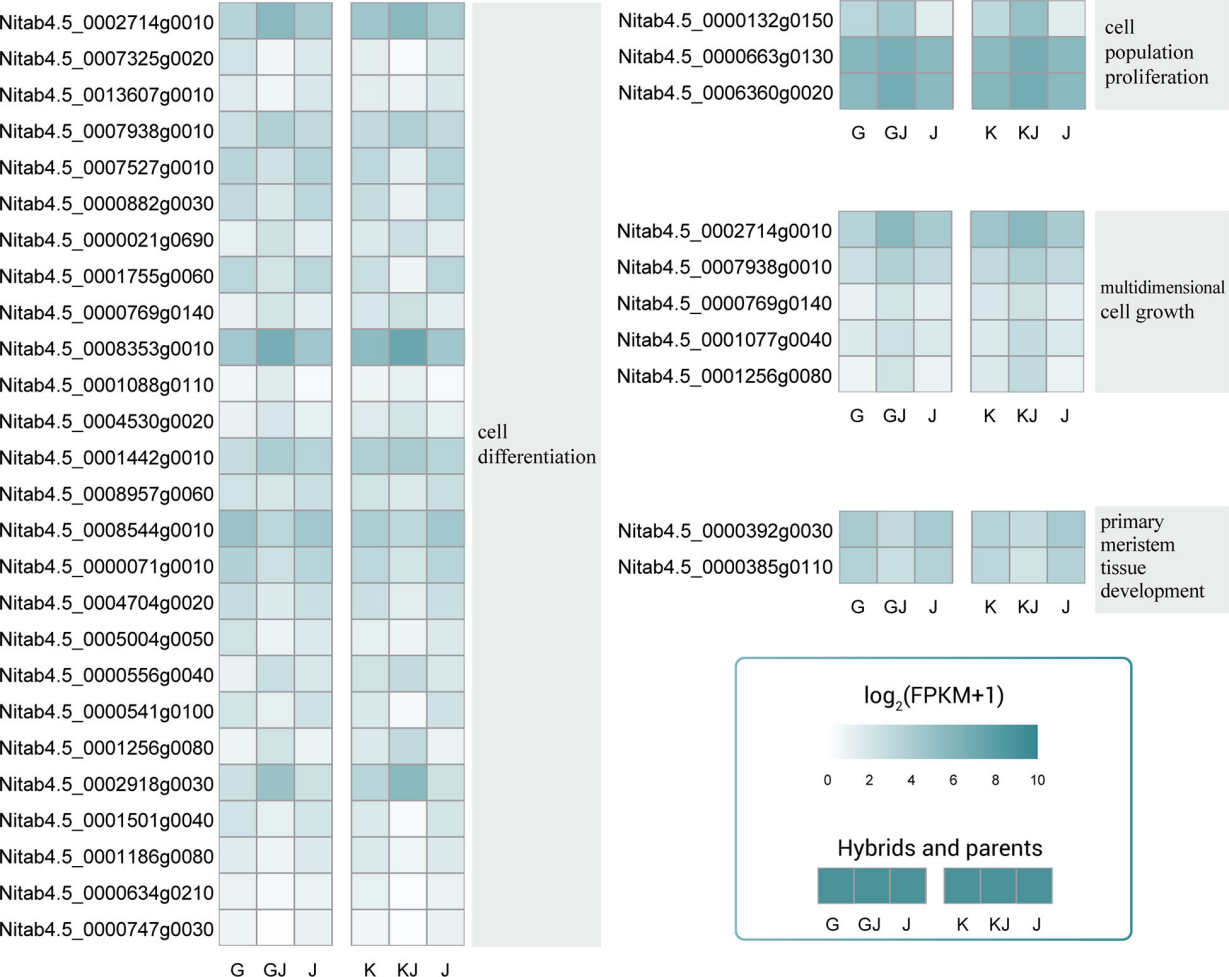
Overdominant expression genes related to cell population promotion, cell differentiation, multiple cell growth and primary commodity issue development. These genes were overdominant expression in both hybrids.

Among the common overdominant expression genes of the two hybrids, various GO categories such as cell population promotion, cell differentiation, multiple cell growth and primary meristem tissue development are significantly enriched. In the process of cell differentiation, most genes show up-regulated overdominant expression (**Figure 7**), in which *Nitab4.5_ 0002714g0010*、*Nitab4.5_ 0008353g0010*、*Nitab4.5_ 0002918g0030* showed high expression in hybrids. In the process of cell population promotion and cell population promotion, 3 and 5 genes were up-regulated overdominant expression in the hybrid, indicating that cell population promotion and cell population promotion were important processes of root heterosis. In the process of primary meristem issue development, the two genes are both expressed down-regulated in the hybrid. In general, a lot of changes have taken place in cell population promotion, cell differentiation and multiple cell growth of hybrids, which may be the reason for the formation of the heterosis of root.

### Identification of gene co-expression network modules

**Figure 8:**
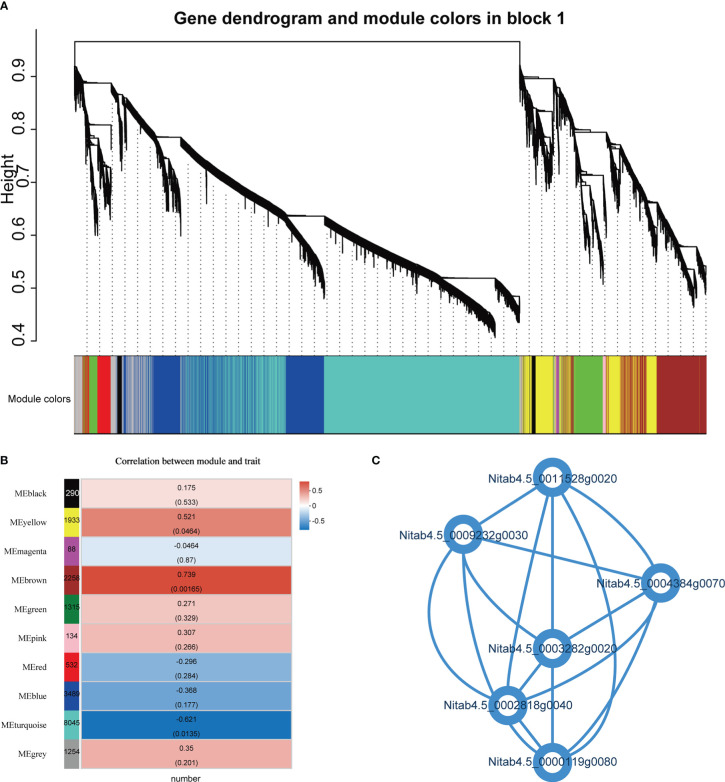
Weighted gene co-expression network analysis (WGCNA). **(A)** Hierarchical cluster tree shows 10 modules of co-expressed genes. The lower heatmap shows modules in designated colors. The module grey is for unassigned genes. **(B)** Heat maps showing the correlation of module-trait, the Pearson correlation coefficient and P values of significant modules are given. **(C)** Connectivity between 6 genes.

A weighted gene co-expression network analysis (WGCNA) was performed on all genes to thoroughly characterize the expression of genes related to root number. WGCNA identified 10 vivid modules (including 19338 genes) (**Figure 8A**; **Supplementary File 1, Table 14**). The module analysis revealed that there was a significant correlation between the brown module and the heterosis of the number of hybrid roots (**Figure 8B**). The genes in the “brown” module are mainly related to cysteine biological process, protein serine/threonine kinase activity, ethylene binding, etc. Further analysis of 6 genes with degree>700 in the brown module, *Nitab4.5_ 0000119g0080* gene is Lysine tRNA ligase, *Nitab4.5_ 0004384g0070*、*Nitab4.5_ 0011528g0020*、*Nitab4.5_ 0003282g0020* gene is Serine/threonine -/dual specificity protein kinase, *Nitab4.5_ 0002818g0040* gene is NAD dependent emergency/dehydratase, *Nitab4.5_ 0009232g0030* gene is Mitochondrial carrier protein (**Figure 8C**). Interestingly, these genes are up-regulated overdominant expression patterns. We believe that these genes may be related to the formation of root heterosis.

### RT-qPCR results

**Figure 9:**
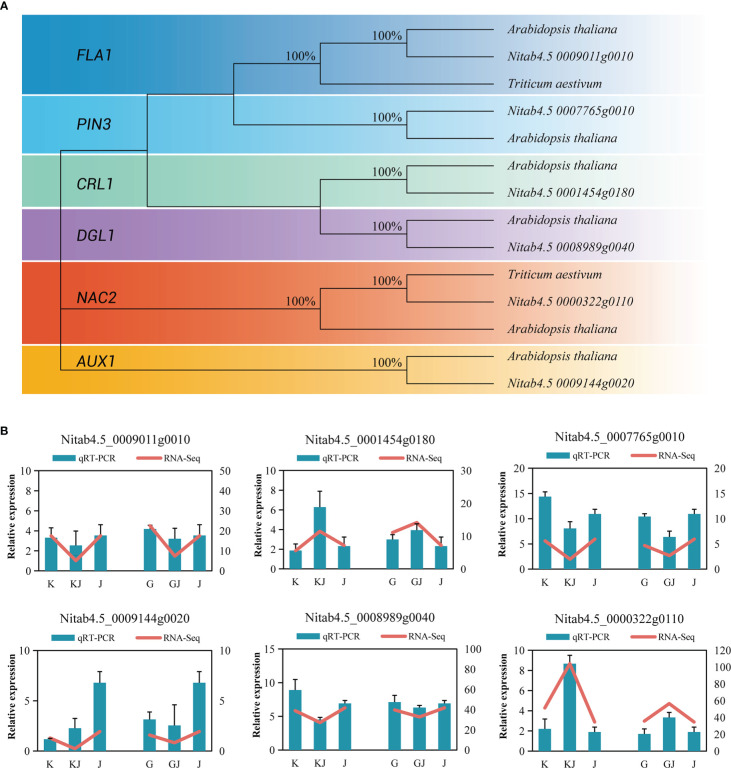
**(A)** Phylogenetic trees of root development genes in tobacco. **(B)** Comparison of RNA-seq and RT-qPCR gene expression levels of root related genes in two hybrids and their parents.

RT-qPCR was used to analyze 6 overdominant genes that have been shown to be involved in root development in other crops. In brief, protein sequences of well-characterized genes of each family from Arabidopsis thaliana were used as BLAST query sequences (**Supplementary File 1, Table 15**), and the following sequences in tobacco were filtered by stringent threshold (E-value <1E-25, % identity>30 and % query coverage>30). In tobacco, protein sequences of root development exhibited a clear topological relationship, with high bootstrap values (Arabidopsis thaliana, Triticum aestivum *L*.)(**Figure 9A**). *Actin* gene was used as a reference gene to standardize the expression levels of each gene. **Supplementary File 1: Table 16** lists the genes and corresponding primers used in the RT-qPCR. The results showed that (**Figure 9B**), the genes of root development also showed a trend of overdominant expression. Although the expression multiples of RT-qPCR data and RNA-seq sequencing results are slightly different, they basically show a consistent expression trend, so RNA-seq data has certain reliability.

## Discussion

Heterosis refers to the superiority of hybrid F_1_ to either parent for a specific trait. The utilization of heterosis has improved crop yield and quality worldwide (Meyer et al., 2004). Many studies have shown that heterosis can be observed in the seedling stage of plants, such as *Arabidopsis* (Zhu et al., 2016; Liu et al., 2020), rice (Ma et al., 2011; Zhu et al., 2016; Liu et al., 2018), corn (Paschold et al., 2012), and wheat (Liu et al., 2018). The application of transcriptome analysis in heterosis research has effectively revealed the molecular basis of heterosis in *Arabidopsis* (Groszmann et al., 2015; Liu et al., 2021), rice (Katara et al., 2020), corn (Song et al., 2013; Guttikonda et al., 2020), cotton (Shahzad et al., 2020), and soybean (Zhang et al., 2017), but the molecular basis of heterosis in tobacco root growth has not been reported in the literature. In the early stage of tobacco growth, improved root development plays an important role in enhancing the absorption of nutrients and water by plants, promoting their growth and increasing their yield (Zhang et al., 2020; Shalby et al., 2021). From this point of view, through comparative transcriptome analysis of roots, the genetic basis of root development heterosis of two strong heterosis hybrids and their parents was studied.

Plant hormones are signal molecules produced by plants, which are used to regulate various aspects of plant growth and development (Santner et al., 2009). Plant hormone signals are sensed and transmitted to the nucleus through a series of signal transduction elements which regulate gene expression and trigger a series of physiological processes. Through the KEGG pathway enrichment analysis of the overdominantly expressed genes, we found that the overdominant genes encoding hormone signal transduction between F_1_ hybrids and parents were significantly enriched. In order to reveal that hormone signal transduction is involved in the growth and development of tobacco roots, we focused on AUX, CTK, ABA, ET, and SA.

Auxin, including biosynthesis and transport of AUX and its signaling, plays a crucial role in regulating root growth and development (Saini et al., 2013). A total of 11 overdominant expression genes (ODEs) encoding AUX signal components in the two hybrids were identified in this study, including 3 *AUX1* genes, 5 *GH3* genes, and 3 *SAUR* genes. *GH3*s and *SAUR*s are AUX early response genes (Yang et al., 2015; Stortenbeker and Bemer, 2019). With 8 of the 11 ODEs being AUX early response genes, this shows that AUX plays a core role in regulating the growth and development of tobacco roots.

CTK regulates many developmental processes of plants, including chloroplast formation, root growth, and nutrient absorption (Zubo and Schaller, 2020). *A-ARRs* have been reported as negative regulators of the CTK signaling pathway (To et al., 2004; Kieber and Schaller, 2014), inhibiting CTK signaling, whereas CTK receptor *CRE1* plays an important role in regulating lateral root development (Gonzalez-Rizzo et al., 2006). In our study, *CRE1* was shown to be down-regulated overdominant and type *A-ARRs* were up-regulated overdominant, which indicated that CTKs accumulated less in the roots of the hybrid F_1_ generation.

Abscisic acid signaling is also important during root development (Brookbank et al., 2021). Some studies have shown that ABA is a negative regulator of lateral root appearance, but genetic evidence also suggests that ABA and AUX have a regulatory interaction in lateral root formation (Wang et al., 2019). *SnRK2* has been reported to organize root development under non-stressful conditions and is necessary in all root tissues (Kawa et al., 2020). Overexpression of *NtSnrk2.2* increased soluble sugar accumulation, increased lateral roots, and improved root development in tobacco plants (Liu et al., 2020). Group A protein phosphorase 2Cs (*PP2Cs*) are ABA co-receptors that negatively regulate the ABA signaling pathway by inhibiting the downstream *SnRK2* protein kinase. 9 ODEs encoding for ABA signaling were found in the two hybrids.

Ethylene is another important plant hormone. It has been reported that ET stimulates the root growth of many plant species (Qin et al., 2019). Qin and Huang, (2018) reported that ET regulates the root development by changing the synthesis of AUX in *Arabidopsis thaliana*; jasmonic acid and ET jointly regulate AUX signaling and promote root development (Xu et al., 2020). The current results show that 12 ODEs in the two hybrids jointly encode AUX signal transduction, including 4 *ETR* genes (3 were AUX1 genes), 3 *EIN3* genes, 3 *EBF1/2* genes, and 4 *ERF1/2* genes. Among the 12 ODEs, 4 ODEs encoding *ERF1/2* were found to be highly expressed, indicating that *ERF1/2* may play a role in regulating root growth and development.

Salicylic acid was identified as the sixth plant hormone in 1992 (Raskin, 1992). In recent years, a large number of studies have confirmed the important role of SA in plant root genesis (Bagautdinova et al., 2022). *Arabidopsis* hybrid varieties with sub optimal and super optimal SA content show root growth heterosis (Zhang et al., 2016), and chromatin remodeling reduces DNA methylation 1 (*DDM1*), linking heterosis with endogenous SA levels. In our study, the genes for salicylic acid synthesis showed overdominant expression. In the future, we can further study the effect of salicylic acid on the formation of root heterosis.

A weighted gene co-expression network analysis (WGCNA), including three genes Nitab4.5_ 0011528g0020、Nitab4.5_ 0003282g0020、Nitab4.5_ 0004384g0070 has a serial threonine/tyrosine protein kinase catalytic domain. The results showed that this domain affected root development of Arabidopsis thaliana through microtubules in primary root zone cells (Sheremet et al., 2010). *OsESG1* gene in rice regulates the initiation and development of crown and root by controlling auxin response and distribution. The gene also has a Serine threonine/tyrosine protein kinase catalytic domain (Pan et al., 2020). It is worth noting that Nitab4.5_ 0003282g0020 gene has a cysteine rich plant receptor like kinase domain. Research shows that *CRK28* in Arabidopsis plays an important role in root growth and epidermal cell differentiation (Pelagio-Flores et al., 2019). Therefore, we believe Nitab4.5_ 0011528g0020, Nitab4.5_ 0003282g0020 and Nitab4.5_ 0004384g0070 may be the key gene regulating the root growth of hybrid.

## Conclusions

In this study, universal heterosis in the root system of hybrids was proved by measuring the number of roots. Comparative transcriptome analysis of hybrids and their parents showed that heterosis was related to the overall gene expression pattern. GJ and KJ hybrids had 5.08% and 10.87% DEGs compared with their parents, respectively; 84.22% and 90.25% of these differential genes showed an overdominant expression pattern, respectively. Through the GO function enrichment analysis of overdominantly expressed genes, it was found that the “plant hormone signal transduction”, “phylpropanoid biosynthesis”, “MAPK signaling pathway plant”, and “starch and supra metadata” pathways were related to root development. We focused on analyzing the biosynthetic pathways of AUX, CTK, ABA, ET, and SA and showed that the overdominantly expressed genes of these hormone signal transduction pathways may enhance root development in hybrids. Cell population promotion, cell differentiation and multiple cell growth in hybrids may be the reason of root heterosis. In addition, *Nitab4.5_0011528g0020、Nitab4.5_0003282g0020、Nitab4.5_0004384g0070* may be genes involved in root growth. To sum up, this study provides a new understanding of the relevant mechanisms of tobacco root heterosis formation. However, further gene function studies are needed to elucidate the development of tobacco root heterosis.

## Data availability statement

The datasets presented in this study can be found in online repositories. The names of the repository/repositories and accession number(s) can be found below: https://www.ncbi.nlm.nih.gov/, https://www.ncbi.nlm.nih.gov/geo/query/acc.cgi?acc=GSE218712.

## Author contributions

KP planned and designed the research, analyzed the data, and wrote the manuscript. WL and SZ performed most of the field work; ZM and LD carried out the molecular biology studies. RL and YH conceived the study and participated in the design and coordination. All authors reviewed the manuscript. All authors contributed to the article and approved the submitted version.
